# The need for understanding and engaging the patient as consumer of products developed by neural engineering

**DOI:** 10.1088/1741-2552/aac668

**Published:** 2018-05-21

**Authors:** Jennifer French, Dawn Bardot, Emily Graczyk, Allison Hess-Dunning, J Luis Lujan, Megan Moynahan, Winny Tan, Ronald Triolo, Adeline Zbrzeski

**Affiliations:** Neurotech Network jfrench@neurotechnetwork.org; Medical Device Innovation Consortium; Case Western Reserve University; Advanced Platform Technology Center, Louis Stokes Cleveland Veterans Affairs Medical Center; Mayo Clinic; Case Western Reserve University; iMEC Corporation; Advanced Platform Technology Center, Louis Stokes Cleveland Veterans Affairs Medical Center Case Western Reserve University; Synapse Biomedical

## To the Editor,

Neural Engineering is a discipline at the intersection of neuroscience, engineering, and clinical care. Recent major efforts by government and industry aimed at bringing forth personalized therapies, increasing the potential of the neural engineering industry for future growth, including the National Institutes of Health (NIH) Brain Research through Advancing Innovative Neurotechnologies (BRAIN) Initiative and Stimulating Peripheral Activity to Relieve Conditions (SPARC) Common Fund Program, the Defense Advanced Research Projects Agency (DARPA) Electrical Prescriptions (ElectRx) and Systems-Based Neurotechnology for Emerging Therapies (SUBNETS) Programs, and the GlaxoSmithKline Bioelectric Medicines Initiative. However, the incremental development of neural technologies can easily become a case of advancing technology for its own sake. This mindset can lead to a solution looking for a problem, without taking into consideration the patient/consumer point of view.

The 2017 Neural Engineering Workshop (NEW) held in Cleveland, Ohio brought together neural engineering leaders. They identified opportunities and challenges to improve the lives of people living with neurological disorders or injury and advance neurotechnology (e.g. neuromodulation and neuroprosthetic fields). During deliberations of the interactive sessions, key stakeholders affirmed the need for the neural engineering community to take a scientific approach to gaining input from patients as consumers to inform the development of neurotechnology devices [1].

*A Roadmap for Neural Engineering* was published after the 2015 NEW meeting containing theme areas including innovation and translation, reimbursement, regulatory, funding resources, clinical practice and patient as a consumer. The consensus from the 2017 NEW participants was a need to not only better understand the consumer, end-user, or patient of neural engineering technologies, but to gain this understanding in a scientific manner. To achieve this, the first step is to clarify objectives using the needs statement format. With collaborative discussions, workshop participants crafted a series of statements to guide the deeper understanding of the potential user of a neural device in her/his daily life. The key identified needs statements are:

1. Build a mechanism through which individuals from the intended population communicate medical and daily living needs free-from-observer bias in order to define, prioritize, and refine development efforts resulting in patient-centric product requirements.
2. Create methods by which the neural engineering community can identify the key needs of a target population in order to validate proposed solutions while maintaining engagement of all stakeholders.
3. Develop a framework to facilitate and grow a collective voice of neurotechnology product consumers and use it to influence stakeholders along the development and care pathway in order to apply evidence-based consumer perspectives.
4. Build a process within the current environment to address patient expectations and considerations when neurotechnology product or study discontinuation occurs in order to reduce secondary loss of function and/or associated mental health.

The neural engineering thought leaders anticipate these identified gaps will guide future patient as consumer engagement. By incorporating these key statements at every stage of the neurotechnology design process, we will ensure that the patient, and not technology, is the focus of development. In turn, ensuring that neural engineering therapies reach the intended populations for maximum therapeutic efficacy and improved quality of life while sustaining the growth of the neural engineering industry.
